# Beta-Cell-Derived Extracellular Vesicles: Mediators of Intercellular Communication in the Islet Microenvironment in Type 1 Diabetes

**DOI:** 10.3390/cells13231996

**Published:** 2024-12-03

**Authors:** Mette C. Dekkers, Xudong Pu, Agustin Enciso-Martinez, Arnaud Zaldumbide

**Affiliations:** 1 Department of Cell and Chemical Biology, Leiden University Medical Center, 2333 ZA Leiden, The Netherlands; m.c.dekkers@lumc.nl (M.C.D.); x.pu@lumc.nl (X.P.); a.enciso_martinez@lumc.nl (A.E.-M.); 2 Oncode Institute, 3521 AL Utrecht, The Netherlands; 3 Amsterdam Vesicle Center, Biomedical Engineering and Physics and Laboratory of Experimental Clinical Chemistry, Amsterdam UMC, University of Amsterdam, 1105 AZ Amsterdam, The Netherlands

**Keywords:** type 1 diabetes, ER stress, extracellular vesicles, inflammation

## Abstract

Type 1 diabetes (T1D) is a chronic autoimmune disorder characterised by an autoimmune response specifically mounted against the insulin-producing beta cells. Within the islet, high cellular connectivity and extensive vascularisation facilitate intra-islet communication and direct crosstalk with the surrounding tissues and the immune system. During the development of T1D, cytokines and extracellular vesicles released by beta cells can contribute to the recruitment of immune cells, further amplifying autoimmunity and aggravating beta cell damage and dysfunction. In this review, we will evaluate the role of beta-cell-derived extracellular vesicles as mediators of the autoimmune response and discuss their potential for early diagnosis and new therapeutic strategies in T1D.

## 1. Introduction

The global incidence of type 1 diabetes (T1D) has been consistently rising, currently affecting close to 9 million people worldwide and predicted to affect 13.5–17.4 million people by 2040 [1]. T1D develops over time, with the progressive dysfunction and destruction of pancreatic beta cells, such that, by the time of clinical manifestation, affected individuals have lost a substantial portion of their functional beta cell mass [2]. T1D is characterised by autoimmune β-cell destruction, leading to insulin insufficiency and elevated blood glucose levels. Persistent hyperglycaemia leads to the development of life-threatening diabetes-associated complications such as blindness, stroke, kidney disease, and heart disease, thus decreasing the quality of life of patients and imposing a considerable economic burden on society [3]. A century after the discovery of insulin, the limited understanding of the molecular mechanisms behind the destruction of insulin-producing cells in the pancreas is hindering the development of new therapeutic strategies. While impaired thymic education or low-affinity T-cells are believed to be responsible for the immune attack directed against native self-proteins, increasing evidence suggests that local inflammation or other types of stress combined with genetic predisposition may trigger autoimmunity through the generation and the accumulation of aberrant or modified proteins to which central tolerance is lacking [4]. In early-stage insulitis, inflammation contributes both to the induction of the immune attack, as well as the functional suppression and apoptosis of beta cells [5]. The communication between immune cells and beta cells is affected by T1D genetic risk variants, including genes encoding for HLA, insulin, PTPN22, PTPN2, and IFIH1 [6]. In this context, islet inflammation and beta cell stress may be triggered by a combination of factors, including genetic predisposition but also other environmental factors such as early-life metabolic changes [7], enteroviral infections [8,9], a high demand for insulin production, or oxidative stress, and is mediated by putative immunogenic signals delivered by other stressed or apoptotic beta cells, cytokine release, and the presentation of neoantigens to the immune system. In addition to these secreted molecules, extracellular vesicles (EVs) play a key role in this communication process and may represent important mediators of T1D pathogenesis.

Accumulating evidence suggests a close link between cellular stress and the secretion of EVs [10]. Several studies have shown that ER stress can increase the release of EVs via the induction of autophagy [11,12]. This self-degradative process represents one of the intrinsic mechanisms to restore cellular homeostasis and is crucial for ensuring survival. The mechanism involves the selection of cytoplasmic macromolecules and organelles for lysosome-mediated degradation. Hence, degraded materials are either released into the cytoplasm for recycling or expelled from the cell. During this process, autophagosomes fuse with multivesicular bodies (MVBs) to form amphisomes, which release their content into the extracellular space by fusion with the plasma membrane. In a state of prolonged ER stress, the unfolded Protein Response (UPR) can promote autophagy through the transcription of autophagy-associated genes, and the Akt1-mTOR and AMPK pathway [13,14]. In addition, the PERK and IRE1 branches of the UPR have been shown to increase the formation of MVBs and stimulate the secretion of exosomes [12]. During the development of T1D, beta cell stress and altered autophagy may influence the formation and secretion of EVs [15]. Functional studies on type 1 susceptibility genes have demonstrated a link to defective autophagy for variants in CLEC16A, encoding for a E3 ligase, shown to mediate protective autophagy, and for CTSH encoding cathepsin H (lysosomal cysteine protease) [16,17].

Although increasing evidence is suggesting a prominent role for EVs in the communication between beta cells and cells of the immune system, showing that beta-cell-derived EVs can modulate immune cell activity [18], a lot remains to be learned regarding the role of these vesicles during T1D disease progression. In cancer, the role of EVs has been studied in greater depth. Extensive research has discovered that the tumour-promoting capabilities of EVs extend beyond the modulation of the local immune response, revealing a role in tumour growth, angiogenesis, epithelial-to-mesenchymal transition, pre-metastatic niche formation, drug resistance, and immune cell polarisation and activation [19,20,21]. Molecular studies have revealed that EVs can contain growth factors, extracellular matrix (ECM) remodelling factors, angiogenic factors, immune evasion molecules, and factors promoting metabolic reprogramming [22]. Learning about the impact of EVs in the tumour microenvironment might help to identify the gaps in our knowledge about how EVs might potentially influence the environment of beta cells in the pancreatic islets in T1D and may offer opportunities for new therapeutic strategies.

## 2. Extracellular Vesicles

EVs are cell-derived particles with a lipid membrane. EVs can be classified based on several criteria including their origin, biogenesis, and size. Exosomes (30–150 nm) originate from the endosomal pathway. In endosomes, intraluminal vesicles are formed through the inward budding of the endosomal membrane, giving rise to MVBs. Here, cargo internalisation is mediated by the Endosomal Sorting Complexes Required for Transport (ESCRT) complex, syntenin, tetraspanins, and ceramide [23]. Upon maturation, Rab and RaI GTPase, SNARE proteins, and syntaxin mediate the docking of MVBs to the plasma membrane, and intraluminal vesicles to ensure the release of exosomes [24].

In contrast to exosomes, ectosomes (150–1000 nm) are derived from the outward budding of the plasma membrane. Their exact biogenesis pathway is not fully understood, but is likely dependent on cytoskeletal components, motor proteins, and fusion machinery [25]. The contents and functions of ectosomes are similar to those of exosomes and are affected by various intrinsic and extrinsic factors, including cellular activation, stress, and signalling pathways.

**Figure 1:**
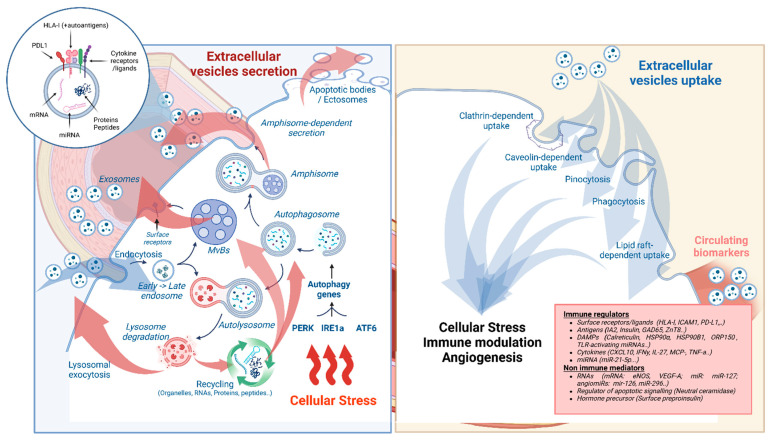
Beta-cell-derived extracellular vesicles: Proposed biogenesis and uptake. Exosome biogenesis is initiated via the endosome pathway and the formation of multivesicular bodies (MVBs). MVBs are essential in cargo sorting, internalisation of cellular components (RNAs, proteins), and recycling of lysosomal degradation products. Ectosomes result from direct budding of the plasma membrane. Upon ER stress and activation of the unfolded protein response, the induction of autophagy acts as an additional adaptive mechanism, forming amphisomes by fusion of autophagosomes with MVBs. Cellular uptake is mediated by lipid-raft or clathrin structures at the cell surface or by membrane invagination (pinocytosis or phagocytosis). Such processes may participate in the amplification of the stress response and the activation of immune cells. Several molecules, and immune and non-immune mediators, specifically derived from beta cells, have been identified and may serve as biomarkers for disease progression.

Apoptotic bodies are relatively large vesicles (1000–5000 nm) that originate from the shedding of plasma membrane vesicles during apoptosis. The formation of apoptotic bodies starts with membrane blebbing, a process driven by cytoskeletal rearrangements and changes in membrane dynamics. These apoptotic blebs continue to grow and engulf cytoplasmic, as well as nuclear, content. Eventually, the blebs become detached from the dying cell and are released into the extracellular space [26] (Figure 1).

The EV cargo is composed of bioactive molecules including proteins, lipids, metabolites, and nucleic acids. Various types of RNAs are present in EVs, but miRNAs and long non-coding RNAs (lncRNAs) are by far the most abundant. Interestingly, there is evidence for the selective sorting of miRNAs in EVs, since miRNAs found in EVs are not identical to miRNAs found in cells [27]. Through the transfer of miRNA and lncRNA, EVs are key players in the post-transcriptional regulation of the gene expression in recipient cells. Although more research on RNA-sorting mechanisms is needed, the manipulation of these pathways may be a new avenue with which to interfere with deleterious EV-mediated intercellular communication.

Intercellular communication is not only mediated by the EV cargo, since the EV surface, composed of lipids, protein, nucleic acids, and glycans, represents a large, highly interactive area. The presence of signalling molecules that are integral or associated with the EV membrane (receptors, transporters, immunoglobulin proteins, MHC molecules, nucleic acid binding proteins, and enzymes [28]) allows EVs to home, target, and activate signalling pathways in recipient cells without uptake. For example, tumour-derived EVs expressing inhibitory ligands MICA, PD-L1, FasL and TRAIL were able to modulate the immune responses of CD8+ T cells and NK cells [22]. Moreover, EV-membrane-bound cytokines play a significant role as they are more stable than cytokines in their free form. Dendritic cell (DC)-derived EVs carrying TNFα on their membrane were able to stimulate NF-κB signalling in HUVECs, demonstrating that EV-tethered cytokines are functionally active [29].

The uptake of EVs by cells occurs via endocytosis, phagocytosis, or fusion. The main pathway of uptake is endocytosis, which includes clathrin-mediated endocytosis, caveolin-dependent endocytosis, phagocytosis, micropinocytosis, and lipid-raft-mediated endocytosis. Internalisation is dependent on interactions between proteins and glycoproteins on the surface of EVs and the membrane of the recipient cell. However, the blocking of a single pathway fails to prevent EV entry, demonstrating that multiple pathways are typically active in parallel. In addition, the heterogeneity of EV samples, containing different types of EVs, also significantly complicates studies on uptake pathways [30].

## 3. The Islet Microenvironment

Beta cells are located in the islets of Langerhans, which are endocrine clusters consisting of approximately 60% beta cells, 30% alpha cells, <10% delta cells, <5% gamma cells, and epsilon cells [31]. In human islets, the different endocrine cell types are typically interspersed, with the majority of the endocrine cells neighbouring cells of a different cell type [32]. While glucose is the main stimulator of insulin release, its secretion is tightly regulated by a balance of nutrient stimuli, paracrine interactions, endocrine signalling, and neuronal stimulation [33]. The electric coupling of beta cells is essential for coordinated glucose-stimulated insulin secretion. Following glucose uptake, Ca^2+^ waves and membrane depolarisation are synchronised within the islet through gap junctions, allowing the diffusion of ions and small molecules between beta cells [34]. In addition to electric coupling, intrinsic beta cell dynamics are important for the function of the collective beta cell compartment of the islet. Beta cell subpopulations, including leader beta cells, hub cells, and follower beta cells, seem to indicate that there is a degree of hierarchy and/or functional specialisation, ensuring the optimal performance of the beta cell network [35]. Indeed, the disturbance of Ca^2+^ oscillation patterns and beta cell hub failure are associated with diabetes mellitus, demonstrating the significance of paracrine beta cell interactions [34,36]. The dependence of beta cells on the other endocrine islet cells is illustrated by the role of glucagon, somatostatin, and ghrelin in the regulation of insulin release. Although glucagon is known to counteract many effects of insulin, glucagon can stimulate insulin release under (mild) hyperglycaemic conditions through the glucagon receptor and GLP1R. Alpha cells appeared to be essential in maintaining proper beta cell function, as beta cells lacking the glucagon and GLP1R release significantly lower the amounts of insulin [37,38]. Alternatively, insulin release is directly regulated by somatostatin and ghrelin released by delta cells and epsilon cells, respectively, via binding to the growth hormone secretagogue receptor (GHS-R) and somatostatin receptor (SSTR) [39,40]. Moreover, in vivo, insulin release is greatly potentiated through the incretin-axis, mediated by insulin-stimulating peptides GLP-1 and GIP, which are produced in the intestine postprandially. To ensure nutrient sensing and the rapid release of hormones into the circulation, the endocrine cells are connected to an extensive microvascular network [41].

The islets are surrounded by a basement membrane and a thin layer of interstitial matrix, and, although the islets appear to be structurally separate from the surrounding tissue, interactions between the endocrine and exocrine pancreas are gaining more attention. Increasing evidence suggests that diseases affecting either the endocrine or exocrine pancreas are linked to abnormalities in the other compartment. The observation that half of the T1D patients exhibit exocrine insufficiency [42], while, in turn, pancreatitis can give rise to diabetes mellitus [43], illustrate this interdependence. Although cause–consequence relations and mechanistic studies are challenging to perform, histological findings support the existence of exocrine–endocrine crosstalk. While, initially, it was suspected that the exocrine and endocrine pancreas were alimented by separate arterioles, investigations conducted on pancreatic sections have revealed that acini receive blood directly from islets through the so-called insulo-acinar portal system, suggesting that acinar cells are exposed to higher concentrations of islet hormones, possibly having more pronounced effects [44]. In this context, the absence of insulin’s tropic effects on acinar cells may partly account for the observed association between T1D and the reduced size of the pancreas [45].

Histological analyses of pancreatic sections from donors at various stages of T1D development have revealed both cellular and structural changes in the islet. In addition to a decrease in the number of beta cells, cellular abnormalities including stress, senescence, and mitochondrial oxidative stress are features of T1D. Beta cells participate in the crosstalk with the immune system through increasing HLA class I expression [46], the release of cytokines, including CXCL10, CCL2, and CCL20, and the expression of stimulatory ligands for NK cell receptors [47,48]. In the noncellular compartment of the islet, ECM remodelling and basement membrane degradation take place, facilitating the infiltration of immune cells [49]. The immune cell infiltrate of the pancreatic islet in T1D is composed mostly of CD8+ T cells, and a smaller fraction of B cells, CD4+ T cells, and macrophages [50].

## 4. Stress, Extracellular Vesicles, and Immune Modulation

Cellular stress is associated with increased immunogenicity, due to the increased expression of immunogenic molecules, including stress proteins, DAMPs and neoantigens [10]. Although this phenomenon has been mainly described in the context of cancer, where neoantigens arise from genomic mutations, fusion proteins, and truncated proteins [51], formed spontaneously [52] or as a result of treatment (chemotherapy, radiotherapy, or small molecules) [53], an increase in cellular stress has been associated with the generation of modified self-peptides, aberrant splice isoforms, and heat shock proteins in autoimmune disease (e.g., Systemic Lupus Erythematosus (SLE) and rheumatoid arthritis (RA)) [54,55,56]. In T1D, while alternative splicing and RNA editing were shown to alter the beta cell translatome [57], neoantigens have been identified through the production of defective ribosomal insulin products (DRiPs), alternatively spliced proteins, hybrid fusion peptides (fusion of insulin fragments with chromogranin A or IAPP), and post-translationally modified proteins (citrullinated Grp78 and IAPP) [58].

Interestingly, it seems that the immunogenic phenotype of stressed cells is also recapitulated in EVs, as seen by the increased release of EVs carrying DAMPs, including HMGB1, Hsp70, and histone H3 upon chemically induced stress [59]. In this study, using choriocarcinoma cells, the increased presence of DAMPs was neutralised upon treatment with antioxidant pyrrolidine dithiocarbamate (PDTC), suggesting that their production and incorporation in vesicles may be dependent on the generation of reactive oxygen species.

It is generally accepted that tumour-cell-derived EVs can participate in immune evasion strategies, by reducing the antigen presentation capacity of DCs [60], inhibiting T-cell activation by releasing PD-L1 containing EVs [61,62], or steering the differentiation of macrophages towards a tumour-promoting (M2) phenotype [63]; functional studies have shown that tumour-cell-derived EVs can also contain immunogenic molecules, and facilitate the transfer of tumour-associated antigens and DAMPs to DCs, increasing the cytotoxicity of T cells and NK cells [64,65,66]. Similarly, these molecules have also been identified in EVs in an autoimmune context. In several autoimmune diseases, it has been shown that EVs containing stress-related molecules were able to trigger proinflammatory responses through the activation of TLRs on the plasma membrane of antigen-presenting cells (APCs). For instance, exosomes isolated from the serum of Graves’ disease patients carrying increased amounts of HSP60 and IGF1R could bind to TLR2/3, stimulate the NF-κB pathway, and increase the production of IL-6 and IL-1β in PBMCs [67]. Moreover, EVs isolated from patients with RA contained oxidised phospholipids, a product of oxidative stress, and were able to activate TLR-4, leading to the increased expression of inflammatory cytokines [68]. EVs can also contribute to the formation of immune complexes, carrying antigens associated with antibodies on the surface. EV-immune complexes isolated from the serum of SLE patients correlated with the presence of autoantibodies and complement consumption, indicating a positive association with disease activity [69]. Another fascinating finding was the detection of MHC antigen complexes on the surface of EVs, and their ability to stimulate T cells either through direct interaction or via uptake by APCs (cross-presentation) [70]. Along that line, a recent study reported the presence of functional proteasomes in platelet-derived EVs, which were able to process exogenous ovalbumin, load the peptide on MHC class I, and, subsequently, stimulate the proliferation of OVA-specific CD8+ T cells [71]. These intriguing findings suggest that EVs from stressed cells may contribute to the development of autoimmunity through the increased production of EVs containing DAMPs and other stress-related molecules, and through their ability to present (auto)antigens.

## 5. Beta-Cell-Derived Extracellular Vesicles and Regulation of the Islet Microenvironment in T1D

Reflective of their origin, EVs derived from pancreatic beta cells may contain insulin, C-peptide, GLP1R, and high levels of insulin mRNA [72]. Interestingly, one of the first cargos of beta-cell-derived EVs discovered was the T1D autoantigen GAD65 [73]. Over the years, the presence of other beta-cell-selective autoantigens was confirmed in EVs derived from primary human islets, including insulin, IA-2, and ZnT8, indicating an active role in initiating or intensifying the autoimmune response targeting beta cells [74,75]. Direct evidence was provided by Rutman et al., who showed that human-islet-derived EVs can induce the activation of memory T and B cells and can stimulate the production of GAD65 antibodies in PBMCs derived from T1D patients [76].

The characterisation of beta-cell-derived EVs (Table 1) mainly arises from in vitro assays where beta cells are exposed to cytokine mixtures to mimic the inflammatory islet environment (e.g., IFNγ, IL-1β, and TNF-α). In these studies, the presence of calreticulin, Gp96, ORP150, and CXCL10, as well as enrichment in proteins involved in TNF-α and ICAM-1 signalling was found, indicating a key role for EVs in the activation of the innate and adaptive immune response [74,77,78]. Moreover, in a study investigating the effect of different types of stress (inflammatory, hypoxic, and genotoxic stress) on the composition and function of beta cell EVs, Giri et al. found that inflammatory stress led to an enrichment of TLR7-binding miRNAs in small EVs, the increased export of MCP-1 in small EVs, microvesicles, and apoptotic bodies, and increased levels of IFNγ, TNF-α, MCP-1, and IL-27 in apoptotic bodies. Upon treatment of bone-marrow-derived DCs, apoptotic bodies derived from cytokine-stimulated beta cells upregulated the expression of MHC class II and CD40 [79]. In contrast, others have shown that apoptotic bodies derived from beta cells after UVB irradiation diminished the expression of CD40 and CD86 and decreased the secretion of IL-6 and TNF-α after uptake by DCs, compared to nontreated DCs [80]. However, due to the minimal characterisation of the apoptotic bodies, the underlying molecular mechanism explaining the observed immunosuppressive effects remains unclear. Nevertheless, similar effects were observed in a recent paper showing that EVs from beta cells and human islets treated with proinflammatory cytokines contained increased levels of PD-L1, an immune checkpoint inhibitor for CD8 T cells [81], demonstrating a dual role of the beta cell EV cargo in the modulation of the immune response.

The extensive microvascular network of the islets of Langerhans allows an active intercellular exchange. Several studies have demonstrated that EVs can mediate the paracrine effects between beta cells [33,41,82,83], providing anti-apoptotic signals, via neutral ceramidase transfer packed in exosomes [84,85], or improving beta cell function by the binding of the EV-surface-bound insulin to the insulin receptor on neighbouring beta cells [86,87]. However, beta-cell-derived EVs also impact alpha cells, as indicated by the increase in glucagon expression after the exposure to EVs from the mouse beta cell lines TC1-α and βTC6 [88]. Although paracrine signalling between islet endocrine cell types has been well-documented, the role of EVs remains an area for further investigation. Interestingly, the EV-mediated crosstalk between beta cells and the islet endothelium has emerged as an area of interest for multiple research groups. In 2014, Figliolini et al. showed that human-islet-derived EVs could stimulate endothelial cell angiogenesis and survival. VEGF-A, eNOS, and miRNA, known to promote angiogenesis (angiomiRs miR-126 and miR-296), were identified as cargo molecules [72]. The pro-angiogenic capacity of islet-derived EVs was further demonstrated in another study which showed that the transfer of miR-127 by EVs promoted cell migration and tube formation in islet endothelial cell line MS1 [89]. In vivo, the transplantation of exosomes from MIN6 cells increased the expression of CD31 in islets of STZ-treated diabetic mice, implying increased islet angiogenesis [87]. The pro-angiogenic effects of the beta-cell- or islet-derived EVs has been of particular interest in the field of islet transplantation. In pathogenic conditions, exosomes from beta cells exposed to proinflammatory cytokines negatively affected the survival of recipient beta cells [90], suggesting a role for EVs in the amplification of the beta cell destruction process.

In addition to the role of beta-cell-derived EVs in driving islet inflammation, EVs derived from other cell types in the islet contribute to the EV-mediated crosstalk. Previous research has shown that exosomes from T cells can promote apoptosis and chemokine expression in beta cells via the transfer of miRNAs miR-142-3p, miR-142-5p, and miR-155, presumably creating a positive feedback loop [18,91].

**Table 1:** Molecules identified in beta-cell-derived or islet-derived EVs potentially mediating intercellular communication. * Presence might be (partially) explained by passive carryover of cytokine treatment.

EV Component	Reported Effect on Recipient Cell	References
**T1D Autoantigens**		
Insulin/preproinsulin	Surface-bound preproinsulin activates insulin receptor signalling and primes glucose-stimulated insulin secretion	[72,74,79,86]
GAD65	GAD65 exosome mimetics can induce GAD65-specific T-cell proliferation	[73,74,75]
IA2	n/a	[74]
ZnT8	n/a	[75]
**Cytokines**		
CXCL10	Promotes beta cell dysfunction, antigen presentation, and recruitment of macrophages and CD8+ T cells	[78]
IFNγ *, TNF-α *, MCP-1, and IL-27	Upregulate the surface expression of CD40 and MHC class II and stimulate TNF-α secretion in APCs	[79]
**DAMPs**		
ORP150, calreticulin, HSPA90α, and HSP90β1	Promote the secretion of TNF-a, IL-1β, and IL-6 in DCs	[74,92]
TLR-activating miRNA	Upregulate the surface expression of CD40 and MHC class II and stimulate TNF-α secretion in antigen-presenting cells	[79]
**Receptors**		
TNFR1	n/a	[77]
ICAM-1 and CD44	Anti-apoptotic and pro-angiogenic effect on islet endothelial cells	[72,77]
PD-L1	Suppresses activation and proliferation of CD8+ T cells	[81]
GLP1R	n/a	[72]
**Miscellaneous**		
eNOS mRNA, VEGF-A mRNA, and angiomiRs	Stimulates angiogenesis in islet endothelial cells	[72]
Neutral ceramidase	Protects beta cells against cell death	[84,85]

## 6. Extracellular Vesicles as Biomarkers for T1D Diagnosis and Progression

**Figure 2:**
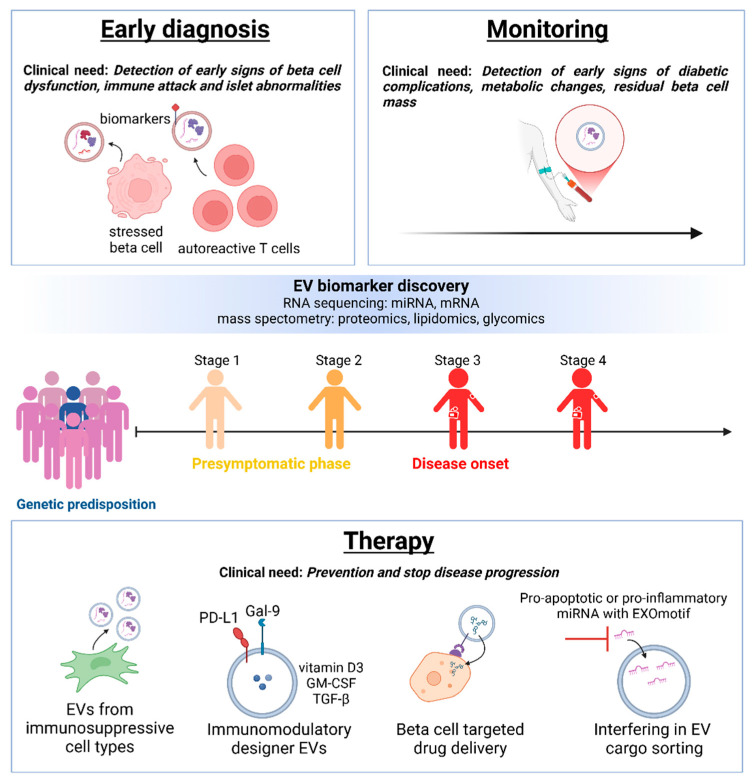
The potential of EVs as a diagnostic, prognostic, and therapeutic tool in T1D. Plasma-circulating EVs may represent a new source of biomarkers for the development and progression of T1D throughout stage 1–4. EVs derived from immunosuppressive cells, such as MSCs, or EVs loaded with immunoregulatory molecules, may be used to inhibit autoreactive immune responses. In addition, EVs may be used as a carrier to facilitate targeted delivery of drugs aimed at restoring the beta cell pool. To reduce pathogenic effects mediated by EV transfer, preventing the incorporation of miRNAs with shared sequences could be a target for a novel therapeutic approach.

The large patient heterogeneity, and the variation in disease progression rates, influenced by factors such as age, BMI, and antibody titers, make individual prognosis challenging [93]. Similar to the cancer field, where EVs have emerged as promising biomarkers [94,95], the identification, characterisation, and detection of the cargo of circulating beta-cell-derived EVs—particularly their RNA content—may aid in the early prediction and monitoring of T1D progression [18] (Figure 2). Several studies have attempted to characterise the EV miRNA cargo after the in vitro exposure of beta cells or islets to pathophysiological conditions and to correlate their expression to circulating levels in the blood from T1D patients.

Recently, using a genetically induced stress model, our group has identified several miRNA characteristics for beta cell stress [96]. In particular, miR-375 was consistently upregulated in the plasma samples of T1D patients [97,98]. Among the other candidates, miR-21-5p was found elevated in serum-derived EVs of diabetic NOD mice. Interestingly, miR-21-5p was elevated 3 weeks prior to diabetes onset. The biomarker potential of miR-21-5p was further supported by the finding that miR-21-5p was three times more abundant in EVs from the serum of children with recent-onset T1D, compared to non-diabetic controls [99]. Interestingly, the same miRNA was enriched in EVs released by beta cell lines and human islets treated with cytokines, and overexpression caused decreased viability via targeting BLC2, indicating that miR-21-5p could be associated with inflammation and beta cell death [100]. Moreover, miR-155-5p may represent a putative novel biomarker. Indeed, its expression is increased in plasma-derived EVs from children with recent-onset T1D and upregulated in beta cells of AAB+ and T1D donors, as demonstrated in pancreatic sections by in situ hybridisation [27]. MiR-155 levels correlated strongly with decreased plasma C-peptide levels, supporting the notion that miR-155 may be directly associated with beta cell dysfunction [101]. Nonetheless, miR-155 has also been found in exosomes from T cells and caused apoptosis in beta cells, suggesting that miR-155 also plays a role in the immune-cell-mediated destruction of beta cells [91]. While an extreme variability has been observed illustrating patient heterogeneity, but, also, EV isolation procedures and sensitivity of the detection methods, several miRNAs were found upregulated. Yet, only a few could be linked to disease progression and beta cell destruction/dysfunction, suggesting that the alteration of the plasma EVs’ miRNA cargo, frequently reported, seems to, rather, reflect an ongoing inflammation or consequent metabolic perturbation than a change in beta cell status.

To investigate the signalling pathways affected by islet-derived EVs, a bioinformatics study was performed on the transcriptome and lncRNA data from human-islet-derived exosomes. The RNA network analysis showed that differentially expressed lncRNAs in EVs from cytokine-stimulated islets were primarily involved in the regulation of the Hippo, TGF-β, Wnt, FOXO, Neurotrophin, and ErbB signalling pathways. Moreover, using other independent datasets for validation, potential biomarkers of T1D were identified, including lncRNA PVT1, LINC00960, and miR-107 [102].

Potential EV-associated mRNA biomarkers were also explored in a study investigating the mRNA profiles of exosomes derived from the plasma of T1D patients. A pathway analysis with the 112 differentially expressed mRNAs revealed that the most significantly enriched terms were positive regulation by the host of viral transcription and oxidative phosphorylation [103]. Of note, viral infections and a type I interferon signature have been associated with T1D risk [104]. Despite these new insights, the role of the identified differentially expressed EV mRNAs in the pathogenesis of T1D and their biomarker potential still requires further investigation. Recently, a systematic review with proteomic data from 13 studies including serum/plasma samples of individuals at different stages of T1D development (pre-seroconversion, post-seroconversion, and post-diagnosis) identified 266 candidate protein biomarkers of T1D, which were linked to pathways related to ECM, cytoskeleton, cellular stress, and immunity [105]. A comparison of the T1D protein biomarker dataset with validated human EV proteins revealed that 41 proteins are represented in plasma EVs. A pathway analysis with the 41 candidates showed the over-representation of the cytokine/chemokine signalling pathway, including branches of the JAK/STAT and SRC/DOCK2/RAC1-2 pathways. Among the other candidates, adhesion molecules, collagen, and MMP-related proteins were found [106]. In inflamed islets, the upregulation of the protease cathepsins and MMPs in macrophages and DCs has been reported, which is suggested to be involved in the degradation of ECM components [107]. Further research will be needed to investigate whether EVs contribute to islet ECM remodeling during the development of T1D. In the future, these data may also be used as a resource for studies aiming at a mechanistic understanding of the role of EVs in T1D disease progression. Since EVs are secreted by all cell types in the human body and carry molecular signatures of the cell of origin, EVs hold great promise for the discovery of biomarkers for disease development. To be of clinical benefit in the management of T1D, there is a need to identify beta-cell-specific EV biomarkers, as well as EV biomarkers related to immune attack and other pathophysiological changes that occur in the islet microenvironment.

## 7. Perspectives and Therapeutical Strategies

While a better characterisation of plasma circulating EVs appears crucial for the selection of robust biomarkers of disease progression, the use of EVs may represent an attractive approach in the treatment of autoimmune diseases.

Exploiting the immunomodulatory properties of EVs has become a novel strategy in therapeutic development. Stem-cell-derived EVs, predominantly from mesenchymal stem cells (MSCs), show great therapeutic promise for T1D due to their intrinsic immunoregulatory and regenerative functions [108,109]. Meanwhile, other groups are pursuing the development of designer EVs. Becker et al. showed that EVs co-expressing HLA-A2, CD80, and PD-L1, produced by modified K562 cells, were able to suppress the PPI-specific T-cell-mediated killing in vitro [110]. However, in vivo studies are needed to investigate whether these EVs can dampen the attack on beta cells. Another group engineered macrophages to generate EVs that overexpress PD-L1 and Gal-9. The administration of PD-L1 and Gal-9 EVs to recent-onset T1D NOD mice reduced the proportion of IFNγ+ and TNFα+ CD8+ T cells and increased the proportion of Foxp3+ Treg cells in the pancreas. PD-L1-Gal-9 EV treatment also significantly improved C-peptide levels and 75% of the mice returned to normoglycaemic range [111]. Alternatively, synthetic biodegradable EVs may present a promising future therapeutic strategy due to their adaptability and scalability. The injection of a combination of synthetic microparticles loaded with insulin B_9–23_ peptide, vitamin D3, TGF-β1, and GM-CSF in NOD mice induced an immunosuppressive phenotype in DCs, increased the PD-1 expression on T cells, and increased Treg induction. Moreover, treatment was able to delay diabetes onset in prediabetic NOD mice and temporally restore euglycaemia in recent-onset NOD mice [112].

Another strategy could involve the control of the EV cargo to selectively blunt beta cell communication with surrounding cells and to limit further beta cell destruction. In this context, while blocking EV uptake using inhibitors of endocytosis, clathrin-mediated endocytosis, or integrin-ligand interactions may potentially result in unwanted effects, interfering in the sorting of specific miRNAs may provide a more targeted approach. Here, the recently identified EXOmotifs associated with the secretion into EVs, rather than cell retention, may represent an opportunity [113].

## 8. Conclusions

Increasing evidence illustrates the important role of the beta cells in provoking the immune system and driving autoimmunity. In this model where the immune system is acting against a defective tissue, in a way that resembles an anti-cancer cell response, beta-cell-derived vesicles are key mediators that can either prevent further destruction and maintain tolerance to beta cell antigens or contribute to the inflammatory process by delivering danger signals and activating immune cells. Cellular stress and inflammation impact the formation, composition, and secretion of EVs, shifting the balance to a predominantly proinflammatory profile by containing autoantigens, cytokines, and DAMPs. Although we have learned a lot on the relation between beta cell EVs and immune cells, our knowledge on their impact on other cell types in the islet microenvironment remains limited.

The diversity of the beta cell vesicular secretome, as well as their cargo and their surface composition, illustrate (i) the complexity of the intercellular communication and the dual role of beta-cell-derived EVs in the context of T1D and (ii) the difficulty of identifying disease biomarkers reflecting beta cell stress/damage in patients. Further research should be aiming at the molecular profiling of EVs derived from in vitro T1D models as well as circulating EVs from patients at different stages of T1D. Although T1D is a heterogenous, multi-organ disease, EVs may contribute to improving disease insight, risk management, and stratification among patients. Using EVs as a therapeutic tool presents many opportunities, including the suppression of the autoimmune response and targeted drug delivery to protect or restore the remaining beta cell mass. Gaining more insight into the cargo-loading mechanisms, targeting RNA-binding proteins and their interactors may be a promising approach with which to intervene in the transfer of proinflammatory or proapoptotic miRNAs to the surrounding tissue and control intra-islet communication.

## Data Availability

No new data were created or analyzed in this study. Data sharing is not applicable to this article.
